# Outcomes of Gallbladder Polyps and Their Association With Gallbladder Cancer in a 20-Year Cohort

**DOI:** 10.1001/jamanetworkopen.2020.5143

**Published:** 2020-05-18

**Authors:** Jean-Luc Szpakowski, Lue-Yen Tucker

**Affiliations:** 1 Department of Gastroenterology, Kaiser Permanente, Fremont, California; 2 Division of Research, Kaiser Permanente, Oakland, California

## Abstract

**Importance:**

Gallbladder polyps (GP) are found in more than 4% of adult abdominal ultrasonographs. Their growth pattern and association with gallbladder cancer (GBC) are poorly defined.

**Objective:**

To determine the growth pattern of GPs and their association with GBC.

**Design, Setting, and Participants:**

This cohort study included 622 227 adult members (ie, aged 18 years or older) of Kaiser Permanente Northern California, an integrated health care delivery system, enrolled between January 1, 1995, and December 31, 2014. The GBC cohort comprised a total of 365 adults with GBC and prior ultrasonography, and the GP cohort comprised 35 970 adults with GPs present on ultrasonography. Data analysis was performed from March 2016 to November 2019.

**Exposures:**

Gallbladder polyps (quantitative size, <6 mm, 6 to <10 mm, and ≥10 mm or qualitative size [ie, tiny, small, moderate, and large]).

**Main Outcomes and Measures:**

For the GBC cohort, proportion of patients with GBC with polyps identified on preceding ultrasonograph. For the GP cohort, rates of GBC among those with polyps according to size and rate of GP growth of at least 2 mm over time.

**Results:**

The GBC cohort comprised 365 individuals (267 [73.1%] women; 173 [47.4%] white patients; median [interquartile range] age, 71 [61-79] years). After excluding 14 patients who did not have evaluation of polyp size, the final GP cohort comprised 35 856 adults, with 18 645 (52.0%) women, a median (interquartile range) age 50 (40-60) years, and 15 573 (43.3%) white patients. Gallbladder polyps were found in 22 patients (6.0%) in the GBC cohort and in 35 870 of 622 227 adults (5.8%) who underwent abdominal ultrasonography. Of these, 19 (0.053%) were diagnosed with GBC, similar to those without GP (316 of 586 357 [0.054%]). The unadjusted GBC rate per 100 000 person-years was 11.3 (95% CI, 6.2-16.3) overall and increased with polyp size, from 1.3 (95% CI, 0-4.0) with initial size of less than 6 mm (n = 17 531) to 128.2 (95% CI, 39.4-217.0) with initial size of 10 mm or larger (n = 2055). In those observed for at least 1 year, the rate was 3.6 (95% CI, 0.7-6.5) per 100 000 person-years. In 6359 patients with evaluable follow-up, unadjusted cumulative probabilities of polyp growth of at least 2 mm at 10 years were 66.2% (95% CI, 62.3%-70.0%) in polyps initially less than 6 mm and 52.9% (95% CI, 47.1%-59.0%) in polyps initially 6 mm to less than 10 mm.

**Conclusions and Relevance:**

In this study, GBC rates were low and similar among patients with and without GPs. Growth of 2 mm or more appeared to be part of GP natural history. The results call into question the strategy of proactively following GP to detect GBC.

## Key Points

**Question:**

How often do gallbladder polyps grow, and are they associated with gallbladder cancer?

**Findings:**

In this cohort study with 622 227 participants aged 18 years or older, growth of gallbladder polyps was common, occurring in 66% of polyps sized less than 6 mm and in 53% sized 6 mm to less than 10 mm. Despite this, gallbladder cancer rarely occurred in those with gallbladder polyps, with an overall rate of 11.3 per 100 000 person-years and, among patients observed for at least 1 year, a rate of 3.6 per 100 000 person-years.

**Meaning:**

The findings of this study suggest that current guidelines recommending periodic ultrasonography of gallbladder polyps to proactively detect gallbladder cancer may need to be revised.

## Teaser

This cohort study assesses the growth pattern of gallbladder polyps and their association with gallbladder cancer.

## Introduction

Gallbladder polyps (GP) are a frequent finding on abdominal ultrasonography, occurring in as much as 4.5% of the adult population.^1,2^ Although most are thought to have no malignant potential, a minority (ie, 4%-10%) are adenomas, which do have malignant potential.^3,4^ Surgical series indicate that the size of polyp is the major risk factor for malignancy, with adenomatous polyps of 10 mm and larger having a 37% to 55% chance of malignancy.^5,6,7^

However, the distinction between adenomas and nonadenomas is usually made after surgery, so if surgery is not performed, the clinician must decide whether to perform surveillance of GP. Studies of the evolution of GP are limited by small sample sizes, with the largest series containing 1553 patients, of whom only 369 (23.8%) had 5-year follow-up.^8,9^ A 2013 American College of Radiology white paper recommended no follow-up for polyps sized 6 mm or smaller and yearly follow-up for polyps sized 7 mm to 9 mm, with surgical consultation if they grow. The duration of follow-up was not addressed.^10^ The 2 studies referenced included 346 patients, of whom 149 (43.1%) underwent ultrasonography follow-up for a mean of 5.4 years,^11^ and 467 patients observed for a median of 39 months.^12^ The American Society for Gastrointestinal Endoscopy also recommended indefinite yearly follow-up for GPs.^13^ A 2017 joint guideline from various European societies recommended surgery for all polyps larger than 10 mm and for those larger than 6 mm with risk factors and 5 years of follow-up for all other polyps. In addition, they stated that polyp growth is rare and recommended surgery for polyps that grow 2 mm or more. The guidelines acknowledged that the recommendations rely on a low level of evidence because of small numbers and short follow-up in the referenced studies.^14^

Given this paucity of data, we undertook a retrospective observational cohort study to answer three questions. First, how often are GPs found on ultrasonographs before diagnosis of gallbladder cancer (GBC)? Second, how often are GPs associated with current or future cancers? Third, how often do GPs grow?

## Methods

### Setting

All participants were adult members (ie, ≥18 years) of Kaiser Permanente Northern California (KPNC), an integrated health care delivery system. The study included members enrolled between January 1, 1995, and December 31, 2014, and data analysis was performed March 2016 to November 2019. During this period, there was no mandatory protocol for follow-up ultrasonographs or cholecystectomies. The Kaiser Foundation Research Institute’s institutional review board approved this observational, data-only study with a waiver of consent because of minimal risk with deidentified data. The study follows the Strengthening the Reporting of Observational Studies in Epidemiology (STROBE) reporting guideline.

### Creation of GBC Cohort

We included all patients with a new diagnosis of GBC and antecedent ultrasonograph in the GBC cohort. Cases were identified through the KPNC Cancer Registry and verified by review of electronic health records. All available imaging data were reviewed to determine whether a GP was detected before cancer diagnosis. For this cohort, the antecedent ultrasonograph or computed tomography scan date was not restricted to the period between 1995 and 2014 and thus the number of patients with GBC differed from the number in the next part of the study.

### Creation of GP Cohort

We identified all KPNC plan members who had an abdominal ultrasonograph performed within our health plan during the study period using *Current Procedural Terminology* codes (ie, 76700 and 76705). We excluded patients with GBC diagnosed before the first ultrasonograph in the study period and those whose initial exam showed an absent gallbladder, as determined by natural language processing. We also excluded those with membership in KPNC for fewer than 30 days after the index ultrasonograph to allow time for follow-up. Ultrasonography was performed according to the standards of the time; contrast enhanced ultrasonography was not performed.

A GP cohort was created with patients whose index date was the first date of an ultrasonograph with GP during the study period. Follow-up was extended through June 30, 2015, with censoring at death, end of plan enrollment, GBC diagnosis, or cholecystectomy. We identified GPs and their sizes from the abdominal ultrasonograph reports using natural language processing with I2E (Linguamatics). Participants were categorized into the 4 following groups based on the initial GP size: less than 6 mm, 6 mm to less than 10 mm, 10 mm or larger, and qualitative size only (ie, tiny, small, moderate, or large). These sizes were chosen because 6 mm is used in guidelines to demarcate different risk groups and 10 mm is used as an indicator of high risk of GBC and therefore an indication to consider cholecystectomy.^10,13,14^ Baseline demographic and clinical data collected at entry to cohort included age, sex, race/ethnicity (ie, white, black, Asian, Hispanic, or other), comorbid conditions (ie, diabetes, hypertension, hyperlipidemia, hypertriglyceridemia), and Charlson Comorbidity Index score. Race/ethnicity was self-reported by patients and was included to assess generalizability of the findings. A non-GP cohort consisted of all patients who had eligible ultrasonographs without GPs detected, with the index date of the first ultrasonograph performed during the study period.

### Outcome Variables

The outcomes for the GP cohort were diagnosis of GBC, polyp growth of at least 2 mm, GP size reaching 10 mm or larger, and cholecystectomy. Crude rates and rates per 100 000 person-years were calculated for the overall cohort and for each initial polyp size group. Cholecystectomies were identified using *International Classification of Diseases, Ninth Revision, Clinical Modification* (*ICD-9-CM*) procedure codes (ie, 51.21-51.24). For the non-GP cohort, the outcome of interest was GBC, with a secondary outcome of cholecystectomy.

Determination of growth was limited to members with at least 2 ultrasonographs with quantitative polyp size. We took growth of at least 2 mm during follow-up to represent true growth rather than measuring error, in accordance with the recommendations of the European radiological, surgical, and endoscopic societies.^14^ Growth of less than 2 mm was defined as stable size. We also assessed the rate of polyps reaching 10 mm. In those with at least 3 quantitative polyp sizes, we determined whether growth of at least 2 mm or reaching at least 10 mm occurred after periods of no growth for 1, 3, and 5 years. Follow-up was until the last evaluable ultrasonograph.

### Statistical Analysis

Categorical baseline characteristics and unadjusted crude GBC rates by group were compared using χ^2^ tests, and nonnormally distributed continuous variables (ie, age at initial polyp and Charlson Comorbidity Index score) were compared using nonparametric Kruskal-Wallis tests. Crude rates of GBC with and without GP were compared with the χ^2^ test. Survival analyses were performed using the Kaplan-Meier method to estimate the survival function (ie, growth of at least 2 mm or reaching 10 mm) among participants with 2 or more quantitative polyp sizes, the probability of cholecystectomy, and the probability of GBC. All these were stratified by initial size groups, and survival was compared using the log-rank test. All analyses were conducted in SAS version 9.4 (SAS Institute). Statistical significance was set at *P* < .05, and all tests were 2-tailed.

## Results

### Rate of GP in the GBC Cohort

We identified 469 individuals with a diagnosis of GBC from 1995 to 2014. A total of 12 (2.6%) were excluded; of those 2 (16.7%) had cancer diagnosed before joining the health plan, 5 (41.7%) had normal gallbladder pathology, 1 (8.3%) had normal subsequent gallbladder imaging without pathology supporting the diagnosis, and 4 (33.3%) lacked supporting data in the electronic health record. Of the remaining 457 individuals, 365 (79.9%; 267 [73.1%] women; 173 [47.4%] white patients; median [interquartile range] age, 71 [61-79] years) had antecedent abdominal ultrasonographs, of whom 22 (6.0%) had polyps diagnosed at some point, including 1 (4.5%) that was seen transiently and 4 (18.2%) that were also described as masses. Of the 325 (71.1%) with computed tomography scans, none showed polyps not already identified by ultrasonography. Overall, 17 cancers (3.7%) were in situ, including 3 of the 22 (13.6%) with polyps. Ultrasonography was performed a median (interquartile range [IQR]) of 7 (1-42) days before diagnosis.

### GP Cohort

**Figure 1.**
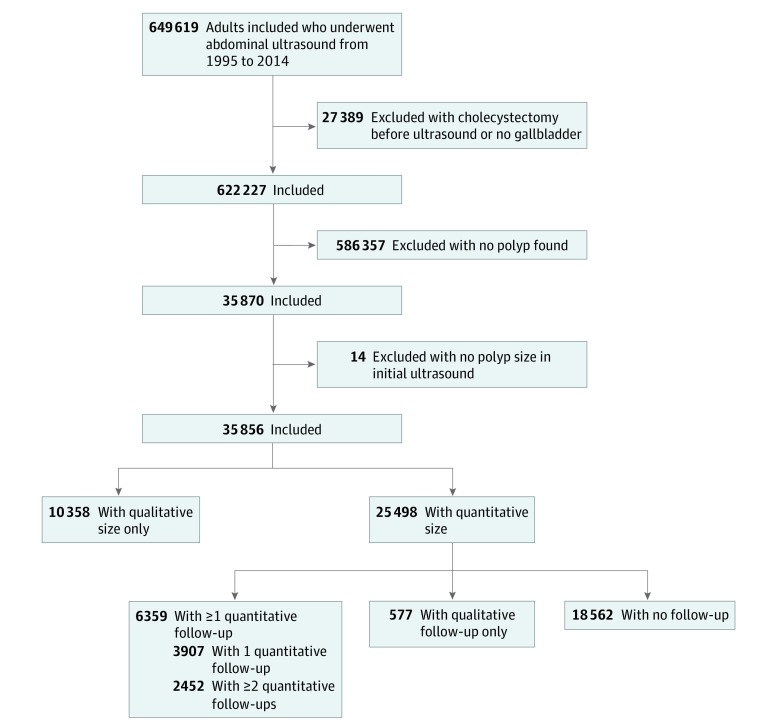
Study Flow Chart

Between 1995 and 2014, 622 227 adults underwent abdominal ultrasonography; GPs were present in 35 870 (5.8%), of whom 14 (<0.1%) had polyps with no recorded size, leaving a final cohort of 35 856 (Figure 1). Baseline demographic and clinical characteristics of the entire GP cohort and by polyp size are shown in Table 1. The cohort included 18 645 (52.0%) women and 15 572 (43.3%) black, Hispanic, and Asian patients and patients with other race/ethnicity, with a median (IQR) age 50 (40-60) years. None of the excluded patients, including those excluded for lack of membership, were diagnosed with GBC as of June 30, 2015.

**Table 1.** Baseline Demographic and Clinical Characteristics of Patients With Gallbladder Polyps^a^

Characteristic	No. (%) by GP size				
	Entire GP cohort (N = 35 856 )	<6 mm (n = 17 531 )	6 to <10 mm (n = 5912)	≥10 mm (n = 2055)	Qualitative size only (n = 10 358)
Women	18 645 (52.0)	9549 (54.5)	2942 (49.8)	992 (48.3)	5173 (49.4)
Age, median (IQR), y^b^	50 (40-60)	49 (39-60)	50 (40-60)	53 (42-64)	51 (41-61)
Age ≥65 y	6465 (18.0)	2892 (16.5)	1045 (17.7)	492 (23.9)	2036 (19.7)
Race/ethnicity					
White	15 573 (43.4)	7520 (42.9)	2493 (42.2)	877 (42.7)	4683 (45.2)
Black	1620 (4.5)	746 (4.3)	207 (3.5)	124 (6.0)	543 (5.2)
Asian	9802 (27.3)	5043 (28.8)	1685 (28.5)	522 (25.4)	2552 (24.6)
Hispanic	5930 (16.5)	2829 (16.1)	1097 (18.6)	385 (18.7)	1619 (15.6)
Other	2931 (8.2)	1393 (7.9)	430 (7.3)	147 (7.2)	961 (9.3)
Diabetes	4759 (13.3)	2114 (12.1)	895 (15.1)	360 (17.5)	1390 (13.4)
Hypertension	11 084 (30.9)	5153 (29.4)	1968 (33.3)	814 (39.6)	3149 (30.4)
Hyperlipidemia	9221 (25.7)	4518 (25.8)	1707 (28.9)	670 (32.6)	2326 (22.5)
Hypertriglyceridemia	654 (1.8)	335 (1.9)	140 (2.4)	45 (2.2)	135 (1.3)
Charlson Comorbidity Index score, median (IQR)^b^	0 (0-1)	0 (0-1)	0 (0-1)	0 (0-1)	0 (0-1)
Charlson Comorbidity Index score ≥2	5636 (15.7)	2557 (14.6)	984 (16.6)	440 (21.4)	1655 (16.0)

Abbreviations: GP, gallbladder polyp; IQR, interquartile range.

^a^ All *P* values of the overall comparisons (among all 4 polyp groups) were <.001 with the χ^2^ test, unless otherwise noted.

^b^ Kruskal-Wallis test.

Nearly three-fourths of patients (25 498 [71.1%]) with polyps had a quantitative size assessment, including 17 531 (68.8%) with GP smaller than 6 mm, 5912 (23.2%) with GP sized 6 mm to less than 10 mm, and 2055 (8.1%) with GP sized 10 mm or larger. Overall, 6359 (24.9%) had at least 1 repeated imaging with polyp size reported, and 2452 (9.6%) had 2 or more repeated ultrasonographs with measured GP (Figure 1). There were small differences in characteristics between those who did and did not have repeated ultrasonographs, but none appeared important. However, given the size of the cohort, they reached statistical significance (eTable 1 in the Supplement).

### GBC in the GP Cohort

A total of 19 of 35 856 adults in the GP cohort were subsequently diagnosed with GBC, including 3 (15.8%) in situ, for an overall rate of 11.3 (95% CI, 6.2-16.3) per 100 000 person-years, increasing with polyp size, from 1.3 (95% CI, 0-4.0) with initial size of less than 6 mm (n = 17 531) to 128.2 (95% CI, 39.4-217.0) with initial size of 10 mm or larger (n = 2055). The unadjusted overall crude proportion of GBC for adults with any GP was 0.053%, similar to that for adults without GP (ie, 0.054%; 316 of 586 357; *P* = .94). Unadjusted rates by initial polyp sizes are shown in Table 2 and eTable 2 in the Supplement, and freedom from GBC over time is depicted in eFigure 1 in the Supplement. After the first year, the rate of GBC in those with initial GP size smaller than 10 mm was 1.05 (95% CI, 0-3.10) per 100 000 person-years. Details of the cancers are presented in Table 3; 12 of 19 (63.2%) were diagnosed in patients with initial polyps larger than 10 mm or described as large, of which 4 (33.3%) were also described as masses. The time between initial ultrasonograph with polyp to diagnosis was a median (IQR) of 113 (47-1153) days. A total of 13 (68.4%) were diagnosed within the first year, likely representing prevalent cancers. Excluding the first year, only 6 cases of GBC (31.6%) were diagnosed, an incidence of 3.6 (95% CI, 0.7-6.5) per 100 000 person-years. After the first year, only 1 case of GBC was diagnosed among those with initial polyp size smaller than 10 mm, despite almost 100 000 person-years of follow-up. Stable polyp size for at least 1 year was documented in 4182 individuals (11.7%), 1 of whom was diagnosed with GBC approximately 11 years later. The small number of GBC cases made a multivariable analysis for cancers not feasible.

**Table 2.** Unadjusted Rates of Gallbladder Cancer in Patients With Previously Identified Gallbladder Polyp

Initial polyp size	Rate per 100 000 person-years by length of follow-up					
	All (N = 35 856)	>1 y (n = 31 709)	>2 y (n = 26 674)	>3 y (n = 22 506)	>4 y (n = 18 836)	>5 y (n = 15 720)
<6 mm	1.3	0	0	0	0	0
6 to <10 mm	8.7	4.5	4.8	5.2	5.8	6.6
≥10 mm	128.2	33.4	35.5	38.4	0	0
Qualitative	12.3	4.7	4.8	3.3	3.4	1.8
Any	11.3	3.6	3.8	3.4	2.2	1.6

**Table 3.** Details of Gallbladder Cancers in Ultrasonography with Prior Polyps

Initial polyp size, mm	Initial ultrasonograph date	Time to cancer diagnosis, mo	Ultrasonography description and pathology notes	Miscellaneous notes
30	2010	1.5	Mass size 3.5 cm vs sludgeball	NA
28	2009	2.7	In-situ carcinoma	NA
26	2007	1	NA	NA
23	2010	0	NA	NA
17	2006	38	NA	Chronic renal insufficiency, dialysis, hypertension, no symptoms
15	2007	2	NA	NA
14	2010	0.5	NA	NA
12	2007	43	Focal in-situ carcinoma	NA
9	2001	135	Polyp was 7 mm size on interval ultrasonography performed February 23, 2004	NA
6	2014	8	NA	NA
3	1999	4.5	In-situ carcinoma	NA
Large	2002	0.03	Large mass–like	NA
Large	2010	2	Large mass–like	NA
Large	2012	10.5	Large mass–like	NA
Large	2006	8.25	Polyp was 43 mm on interval ultrasonograph	Interval ERCP, ERCP pancreatitis, colonoscopy
Small or tiny	2003	3.3	NA	NA
Small or tiny	2011	27.5	Follow-up ultrasonographs on March 21, 2012, with 3 mm polyp and on May 18, 2012 with 5 mm polyp	NA
Small or tiny	2008	58.5	Follow-up ultrasonography on March 21, 2012 found small or tiny polyp	NA
Small or tiny	1996	78.5	NA	NA

Abbreviations: ERCP, endoscopic retrograde cholangio-pancreatography; NA, not applicable.

### Increase in GP Size

**Figure 2.**
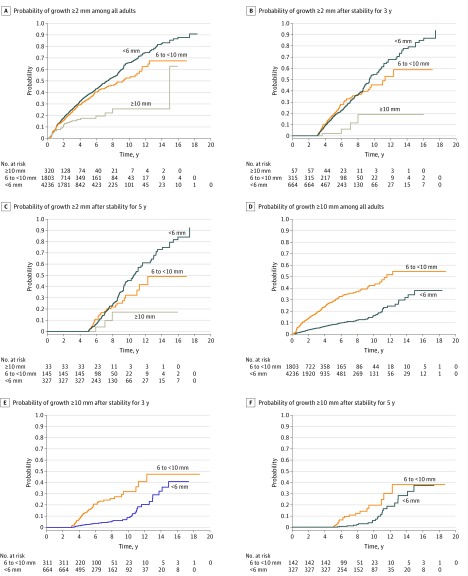
Cumulative Probability of Polyp Growth Kaplan-Meier curves show unadjusted risk of growth of existing gallbladder polyps in adults with quantitative initial polyp size. Follow-up was until the last abdominal ultrasonograph. Probability of growth of at least 2 mm in all adults (A; log-rank tests results for overall comparison: *P* < .001; <6 mm vs 6 to <10 mm: *P* = .01; <6 mm vs ≥10 mm: *P* < .001), after stability for 3 years (B; long-rank test results for overall comparison: *P* = .004; <6 mm vs ≥10 mm: *P* = .03), and after stability for 5 years (C; log-rank tests results for overall comparison: *P* = .03; <6 mm vs ≥10 mm: *P* = .03). Probability of polyp size reaching 10mm in all adults with quantitative initial polyp size of less than 10 mm (D; log-rank *P* < .001), after stability for 3 years (E; log-rank *P* < .001), and after stability for 5 years (F; log-rank *P* = .001).

The cumulative probability of a GP growing 2 mm or more was small but linearly progressive during most of the follow-up period, reaching 66.2% (95% CI, 62.3%-70.0%) in GPs initially smaller than 6 mm and 52.9% (95% CI, 47.1%-59.0%) in those initially sized 6 mm to less than 10 mm (Figure 2A; eTable 3 in the Supplement). Growth was similar for the first 6 years between the groups with an initial polyp size smaller than 6 mm and of between 6 mm and less than 10 mm. The risk of polyps reaching 10 mm also increased linearly over time, with smaller polyps taking longer to reach that threshold (Figure 2D; eTable 3 in the Supplement).

On multivariate analysis, Charlson Comorbidity Index score of at least 2, male sex, and Asian race were associated with at least 2 mm increase in polyp size (Charlson Comorbidity Index score ≥2: hazard ratio [HR], 1.22; 95% CI, 1.02-1.46; female sex: HR, 0.86; 95% CI, 0.78-0.95; Asian race: HR, 1.13; 95% CI, 1.002-1.27), whereas larger polyps were less likely to show measurable growth (initial size ≥10 mm: HR, 0.45; 95% CI, 0.32-0.62) (eTable 4 in the Supplement). For size reaching 10 mm, male sex and Charlson Comorbidity Index score of at least 2 were again associated with increased risk (female sex: HR, 0.75; 95% CI, 0.63-0.89; Charlson Comorbidity Index score ≥2: HR, 1.41; 95% CI, 1.01-1.90), but the greatest risk was associated with larger GPs (initial size 6 mm to <10 mm: HR, 3.79; 95% CI, 3.17-4.52).

Growth to 10 mm was not associated with increased risk of GBC. A total of 507 GPs with at least 1 follow-up ultrasonograph reached a size of 10 mm; of which 210 (41.4%) were initially smaller than 6 mm and 297 (58.6%) were sized between 6 mm and less than 10 mm. In the 1549 person-years of follow-up, no GBC was diagnosed. Of these, 163 (32.2%) underwent cholecystectomies, with definitive pathologic proof of no cancer.

Stable polyp size for 1 year was documented in 4162 individuals (11.6%), for 3 years in 1795 (5.0%), and for 5 years in 880 (2.5%). In those who also had subsequent ultrasonographs (2137 [51.3%] for 1-year stability; 1036 [57.7%] for 3-year stability; and 505 [57.4%] for 5-year stability), the risk of subsequent growth progressed over time parallel with the risk in those without the initial period of stability (Figure 2B and Figure 2C; eTable 3 in the Supplement). After the period of initial stability, the risk of reaching the 10 mm threshold also progressed over time (Figure 2E and Figure 2F; eTable 3 in the Supplement). One patient (<0.1%) whose polyp was stable for 3 years subsequently developed GBC.

### Cholecystectomy

Approximately 1 in 6 patients (5721 of 35 856) with GPs underwent a cholecystectomy in our study period, with only 14 of 5721 (0.2%) showing histological evidence of GBC. The cumulative probabilities of cholecystectomy by size are shown in eFigure 2 in the Supplement. Most cholecystectomies were performed in the first 2 years. For polyps sized 10 mm or larger, the cumulative rate was 37.3% (95% CI, 35.2%-39.5%) at year 2, 39.8% (95% CI, 37.5%-42.1%) at year 4, and 43.9% (95% CI, 41.2%-46.7%) at year 10. The overall proportions of cholecystectomies in patients with and without polyps were 16.0% (5721 of 35 856) vs 19.1% (111 781 of 586 357), respectively (*P* < .001).

## Discussion

We found that 6.0% of KPNC members diagnosed with GBC had GPs on antecedent abdominal ultrasonograph, similar to the 5.8% rate in the general adult population. Furthermore, we found that similar proportions of adults had GBC (0.053% vs 0.054%) whether or not an initial ultrasonograph showed a GP. Both findings suggest that there may not be an overall association of GP with GBC and that GPs are an incidental finding. The exception may be adenomatous polyps, although that association is not yet fully delineated.

Although patients with larger polyps had a higher rate of GBC than those with smaller polyps, the development of GBC was still low in this subgroup. Most GBC was present in the first year, likely representing prevalent cancers. Polyps initially smaller than 10 mm were almost never associated with future GBC, and polyps initially sized 10 mm or larger were only rarely associated with GBC after the first year and not associated after the fourth year (eFigure 1 in the Supplement). This low rate of subsequent cancer did not seem to be associated with the prophylactic removal of gallbladders, given that even for the largest polyps, the cumulative probability of cholecystectomy reached 37.3% at year 2 and increased minimally thereafter (ie, to 43.9% at year 10). The rate of GBC in even larger polyps raises semantic questions, considering that these may be described as masses instead of polyps, as illustrated by a number of our cases.

We found that polyps detected by ultrasonography grew at a low rate, but that growth did not stop over time. Consequently, an increasing percentage reached the 10 mm size threshold over time, which, as expected, happened more commonly with polyps that were initially large. Importantly, this growth did not appear to be associated with future GBC; none of the 507 patients with smaller polyps that grew to 10 mm or larger were subsequently diagnosed with cancer, despite 1549 person-years of follow-up.

Our findings call into question the European societies’ recommendations for follow-up of GP.^14^ Those call for follow-up at 1, 3, and 5 years for polyps smaller than 6 mm and at 6 months and yearly for 5 years for polyps sized between 6 mm and 9 mm. Cholecystectomy is recommended for polyps sized 10 mm or larger and for polyps that grow at least 2 mm. Our study suggests that growth over time is the natural history of GP and that even stability for 5 years does not guarantee subsequent stability. Furthermore, we did not find evidence that this subsequent growth was associated with GBC.

Our findings do support the position of the American College of Radiology that polyps smaller than 7 mm do not need follow-up and suggest that this may also apply to polyps sized between 7 mm and less than 10 mm.^10^ Further studies are needed to validate this approach. A limited strategy of 1 or 2 follow-up ultrasonographs in the subsequent year or 2 years should provide adequate reassurance, if needed. Otherwise the number of yearly ultrasonographs needed to detect GBC is enormous. For example, in polyps smaller than 10 mm, 95 624 ultrasonographs after the first year would be needed to detect 1 GBC (rate, 1.05 per 100 000; range, 0-3.10). For polyps sized 10 mm or larger, cholecystectomy can still be recommended, but the yield in cancers will be low (8 of 2055 [0.4%] in our study), and this should be incorporated into the risk-benefit equation. A fruitful area of research would be determining whether a higher size threshold should be used and the determination of histology in these larger polyps to limit surgery to those with adenomatous polyps.

Our study also raises questions for further studies to confirm and analyze risk factors for growth in GP. Whether the slower growth of larger polyps is real or an artifact of measurement (eg, systematic errors in rounding up or down) is not clear. If studies confirm that Charlson Comorbidity Index score is indeed associated with more growth, the mechanisms (eg, an inflammatory state) remain to be elucidated.

### Strengths and Limitations

Our study has several strengths. To our knowledge, it is by far the largest single study of GP by an order of magnitude, and it far exceeded the total GP population and had a longer follow-up than a 2016 meta-analysis.^8,9^ Our population was multiethnic and representative of the general Northern California population.^15^ We avoided selection bias by including all ultrasonographs regardless of indication performed in our population during the study period, except for the specified exclusions. The follow-up of our population was generally complete. As a prepaid health maintenance organization, there is no incentive for our members to get their care elsewhere; if they do get episodic or emergency care elsewhere, they return to our organization for their routine care, and their diagnoses are captured.

Our study also has several limitations. We only looked at ultrasonograph-detected polyps, with the attendant possibilities of operator errors in interpretation. At the same time, this is a strength because it is the scenario faced by physicians when deciding whether to perform surveillance.

Most follow-up determination of the absence of GBC is based on imaging and clinical criteria. We cannot rule out the presence of GBC that was not detected. However, the length of follow-up argues against that, with 13 236 patients having follow-up of at least 5 years and 4923 of at least 10 years. Furthermore, in 5707 cases we had the ultimate confirmation of absence of GBC, ie, a pathology specimen after cholecystectomy.

It was outside the scope of this study to determine the reasons for performing cholecystectomies in this cohort, and it is unclear how that would change our findings. It was also not possible to capture the indication for ultrasonography because that was not coded, and it is also not clear how indication would affect the natural history of GP.

## Conclusions

Our results suggest that the natural history of GP is to grow over time. Polyps less than 10 mm in size were very rarely associated with GBC and even polyps sized 10 mm or larger rarely did. Most cancers were diagnosed in the first year after polyp diagnosis and were most likely prevalent cancers. The presence or absence of polyps overall did not appear to alter the proportion of GBC. Our study calls into question the strategy of monitoring polyps to proactively detect a GBC. Further studies are needed to address the utility of a higher size threshold to recommend cholecystectomy as well as the practicality and usefulness of determining whether GP are adenomatous.

## Notes

Supplement. **eTable 1. **Characteristics of Gallbladder Polyp Cohort Who Had Follow-up Ultrasonograph Compared With Those Who Did Not **eTable 2. **Rate of Gallbladder Cancer by Initial Polyp Size with Full Raw Data **eTable 3. **Unadjusted Rates of Gallbladder Polyp Size Change in Patients With Gallbladder Polyp **eTable 4. **Risk Factors for Growth of Gallbladder Polyps **eFigure 1. **Gallbladder Cancer Survival in Adults With Gallbladder Polyp **eFigure 2. **Cumulative Probability of Cholecystectomy in Adults With Gallbladder Polyp
