# Supplementary material for: Outcomes of Gallbladder Polyps and Their Association With Gallbladder Cancer in a 20-Year Cohort
Source: JAMA Netw Open. 2020 May 18;3(5):e205143. doi: 10.1001/jamanetworkopen.2020.5143 (PMC7235691; doi:10.1001/jamanetworkopen.2020.5143)
Supplement: Supplement. — eTable 1. Characteristics of Gallbladder Polyp Cohort Who Had Follow-up Ultrasonograph Compared With Those Who Did Not eTable 2. Rate of Gallbladder Cancer by Initial Polyp Size with Full Raw Data eTable 3. Unadjusted Rates of Gallbladder Polyp Size Change in Patients With Gallbladder Polyp eTable 4. Risk Factors for Growth of Gallbladder Polyps eFigure 1. Gallbladder Cancer Survival in Adults With Gallbladder Polyp eFigure 2. Cumulative Probability of Cholecystectomy in Adults With Gallbladder Polyp [file jamanetwopen-3-e205143-s001.pdf]

Supplementary Online Content

Szpakowski J-L, Tucker L-Y. Outcomes of gallbladder polyps and their association with gallbladder cancer in a 20-year cohort. *JAMA Netw Open*. 2020;3(5):e205143. doi:10.1001/jamanetworkopen.2020.5143

eTable 1. Characteristics of Gallbladder Polyp Cohort Who Had Follow-up Ultrasonograph Compared With Those Who Did Not
eTable 2. Rate of Gallbladder Cancer by Initial Polyp Size with Full Raw Data
eTable 3. Unadjusted Rates of Gallbladder Polyp Size Change in Patients With Gallbladder Polyp
eTable 4. Risk Factors for Growth of Gallbladder Polyps
eFigure 1. Gallbladder Cancer Survival in Adults With Gallbladder Polyp
eFigure 2. Cumulative Probability of Cholecystectomy in Adults With Gallbladder Polyp

This supplementary material has been provided by the authors to give readers additional information about their work.

© 2020 Szpakowski J-L et al. *JAMA Network Open.*

eTable 1. Characteristics of Gallbladder Polyp Cohort Who Had Follow-up Ultrasound Compared With Those Who Did Not

All Initial Polyps			
Characteristic	F/U Ultrasound	No F/U Ultrasound	P
Age, year, median [IQR]	49 [40-58]	50 [40-61]	<.0001
Age ≥65 years	12.52 %	19.57%	<.0001
Charlson Comorbidity Index Score, median [IQR]	0 (IQ 0-1)	0 (IQ 0-1)	<.0001
Charlson Comorbidity Index Score ≥2	11.11%	17.00%	<.0001
Female	50.97%	52.33%	0.03
Race/Ethnicity			<.0001
White	36.32%	45.41%	
Black	3.38%	4.84%	
Asian	39.33%	23.99%	
Hispanic	14.43%	17.13%	
Other/Unknown	6.55%	8.63%	
Diabetes Mellitus	11.83%	13.86%	<.0001
Hypertension	27.84%	31.77%	<.0001
Hyperlipidemia	24.85%	25.96%	0.047
Hypertriglyceridemia	1.88%	1.81%	0.68

F/U, follow-up; IQR, interquartile range.
Overall comparisons between groups were tested using χ^2 test for categorical variables and Wilcoxon-Mann-Whitney for nonparametric continuous variables.

© 2020 Szpakowski J-L et al. *JAMA Network Open.*

eTable 2. Rate of Gallbladder Cancer by Initial Polyp Size with Full Raw Data

Initial Polyp Size	Rate/100,000 Person-Years with Different Length of Follow-up					
	Any Follow-up	>1 Year	>2 Years	>3 Years	>4 Years	>5 Years
<6mm						
Sample size	17,531	14,027	11,281	9,065	7,191	5,691
Cancer Cases	1	0	0	0	0	0
Person Years	74,836.2	73,295.4	69,218.8	63,720.0	57,190.4	50,449.8
Rate/100,000 Person-Years	1.3	0	0	0	0	0
95% Confidence Interval	0-4.0	-	-	-	-	-
6-<10mm						
Sample size	5,912	4,376	3,417	2,766	2,199	1,722
Cancer Cases	2	1	1	1	1	1
Person Years	22,917.1	22,329.1	20,912.8	19,302.9	17,322.4	15,178.1
Rate/100,000 Person-Years	8.7	4.5	4.8	5.2	5.8	6.6
95% Confidence Interval	0-20.8	0-13.3	0-14.2	0-15.3	0-17.1	0-19.5
≥10mm						
Sample size	2,055	1,159	911	736	589	477
Cancer Cases	8	2	2	2	0	0
Person Years	6,242.9	5,997.3	5,633.7	5,206.1	4,695.6	4,194.3
Rate/100,000 Person-Years	128.2	33.4	35.5	38.4	0	0
95% Confidence Interval	39.4-217.0	0-79.6	0-84.7	0-91.7	-	-
Qualitative						
Sample size	10,358	8,450	7,510	6,742	6,006	5,346
Cancer Cases	8	3	3	2	2	1
Person Years	64,922.5	64,305.8	62,906.5	60,994.8	58,425.0	55,461.1
Rate/100,000 Person-Years	12.3	4.7	4.8	3.3	3.4	1.8
95% Confidence Interval	3.8-20.9	0-9.4	0-10.2	0-7.8	0-8.2	0-5.3
Any						
Sample size	35,856	28,012	23,119	19,309	15,985	13,236
Cancer Cases	19	6	6	5	3	2
Person Years	168,918.7	165,927.6	158,671.9	149,223.7	137,633.3	125,283.3
Rate/100,000 Person-Years	11.3	3.6	3.8	3.4	2.2	1.6
95% Confidence Interval	6.2-16.3	0.7-6.5	0.8-6.8	0.4-6.3	0-4.7	0-3.8

"-", not applicable

© 2020 Szpakowski J-L et al. *JAMA Network Open.*

eTable 3. Unadjusted Rates of Gallbladder Polyp Size Change in Patients With Gallbladder Polyp

	Crude Rate n/sample size (%)	Rate Per 100,000 Person-Years	Cumulative Probability, %						
			1 Year	2 Years	3 Years	5 Years	6 Years	8 Years	10 Years
Polyp size growth ≥2mm									
<6mm									
Overall	1072/4236 (25.3)	10,047.0	9.1	18.4	25.6	38.4	43.4	53.5	66.2
Stable 3 years	179/664 (27.0)	4728.2	-	-	-	17.0	22.7	37.1	54.3
Stable 5 years	88/327 (26.9)	3563.4	-	-	-	-	6.8	24.2	45.0
6-<10mm									
Overall	373/1803 (20.7)	8605.8	9.0	16.3	23.2	34.7	40.8	46.6	52.9
Stable 3 years	78/315 (24.8)	4507.5	-	-	-	19.4	28.2	37.0	45.5
Stable 5 years	29/145 (0.2)	2720.9	-	-	-	-	10.9	21.7	32.3
≥10mm									
Overall	37/320 (11.6)	4543.0	6.9	12.4	15.5	17.8	19.6	25.9	25.9
Stable 3 years	4/57 (7.0)	1196.9	-	-	-	2.0	5.4	18.0	18.0
Stable 5 years	3/33 (9.1)	1246.3	-	-	-	-	3.5	16.4	16.4
Polyp size reaching 10mm or more									
<6mm									
Overall	210/4236 (5.0)	1830.5	1.8	3.7	5.3	8.3	9.8	12.3	16.3
Stable 3 years	25/664 (3.8)	624.4	-	-	-	2.7	3.6	5.4	8.6
Stable 5 years	9/327 (2.8)	351.4	-	-	-	-	1.1	2.2	5.8
6-<10mm									
Overall	297/1803 (16.5)	6764.7	7.7	13.6	18.5	28.8	32.9	36.9	42.3
Stable 3 years	53/311 (17.0)	3068.8	-	-	-	14.9	20.9	25.6	32.1
Stable 5 years	16/142 (11.3)	1524.3	-	-	-	-	6.4	12.1	19.7

“-“, not applicable.

© 2020 Szpakowski J-L et al. *JAMA Network Open.*

eTable 4. Risk Factors for Growth of Gallbladder Polyps

Adjusted[a] Hazard Risk (95% Confidence Interval) for Growth of Gallbladder Polyps		
	Growth ≥2mm	Growth to 10mm or More
Size 6-<10mm	**0.85 (0.75-0.95)**	**3.79 (3.17-4.52)**
Size ≥10mm	**0.45 (0.32-0.62)**	-
Female	**0.86 (0.78-0.95)**	**0.75 (0.63-0.89)**
Age ≥65	1.03 (0.87-1.21)	1.05 (0.79-1.40)
Black	0.96 (0.71-1.30)	0.89 (0.51-1.57)
Asian	**1.13 (1.002-1.27)**	1.17 (0.96-1.44)
Hispanic	1.12 (0.95-1.31)	1.13 (0.87-1.48)
Other/Unknown	0.94 (0.746-1.19)	0.98 (0.65-1.46)
Charlson Comorbidity Index Score ≥2	**1.22 (1.02-1.46)**	**1.41 (1.04-1.90)**
Diabetes Mellitus	1.10 (0.92-1.32)	0.96 (0.70-1.31)
Hypertension	1.09 (0.96-1.24)	0.89 (0.71-1.12)
Hyperlipidemia	1.10 (0.96-1.25)	1.12 (0.90-1.40)
Hypertriglyceridemia	1.35 (0.96-1.89)	1.23 (0.71-2.15)

[a] Reference groups: initial polyp size <6mm, White.
"-", not applicable.
Bold face indicates hazard rate is statistically significant, 95% confidence interval does not cross 1.

© 2020 Szpakowski J-L et al. *JAMA Network Open.*

eFigure 1. Gallbladder Cancer Survival in Adults With Gallbladder Polyp

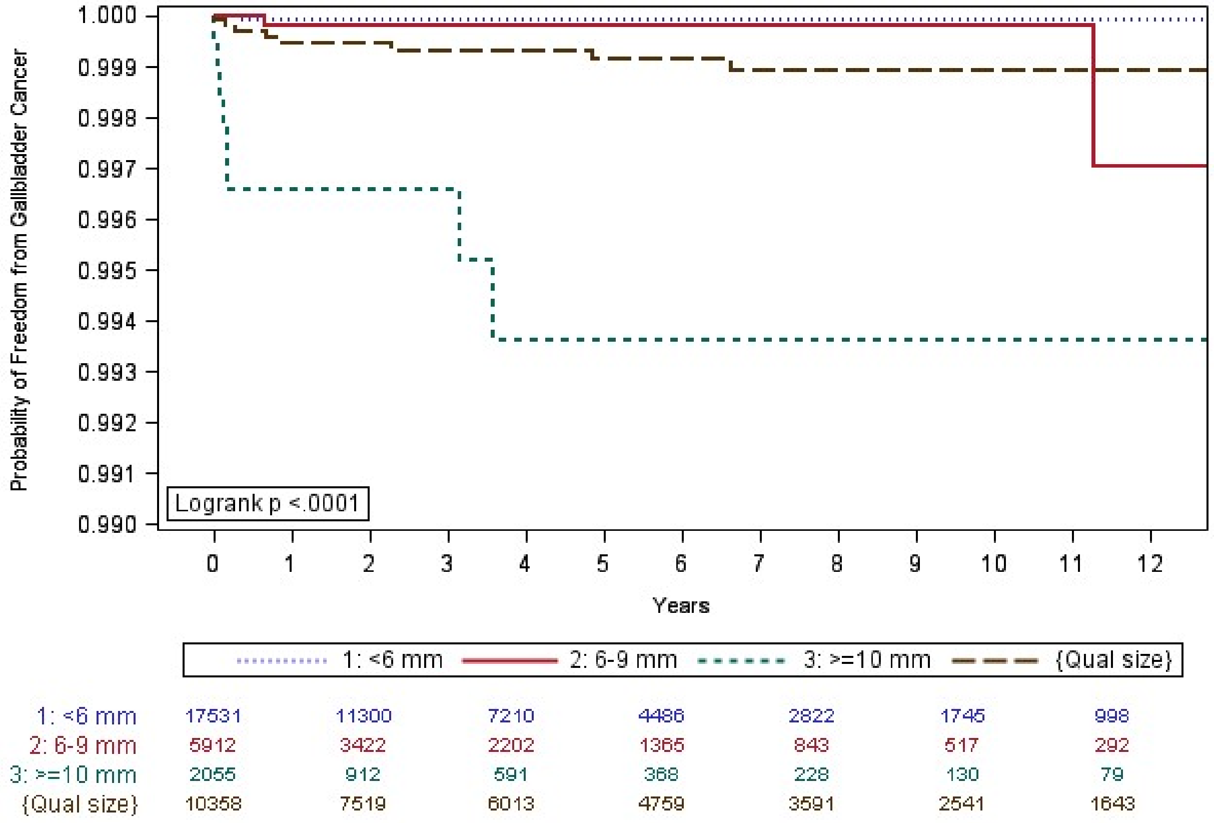

© 2020 Szpakowski J-L et al. *JAMA Network Open.*

eFigure 2. Cumulative Probability of Cholecystectomy in Adults With Gallbladder Polyp

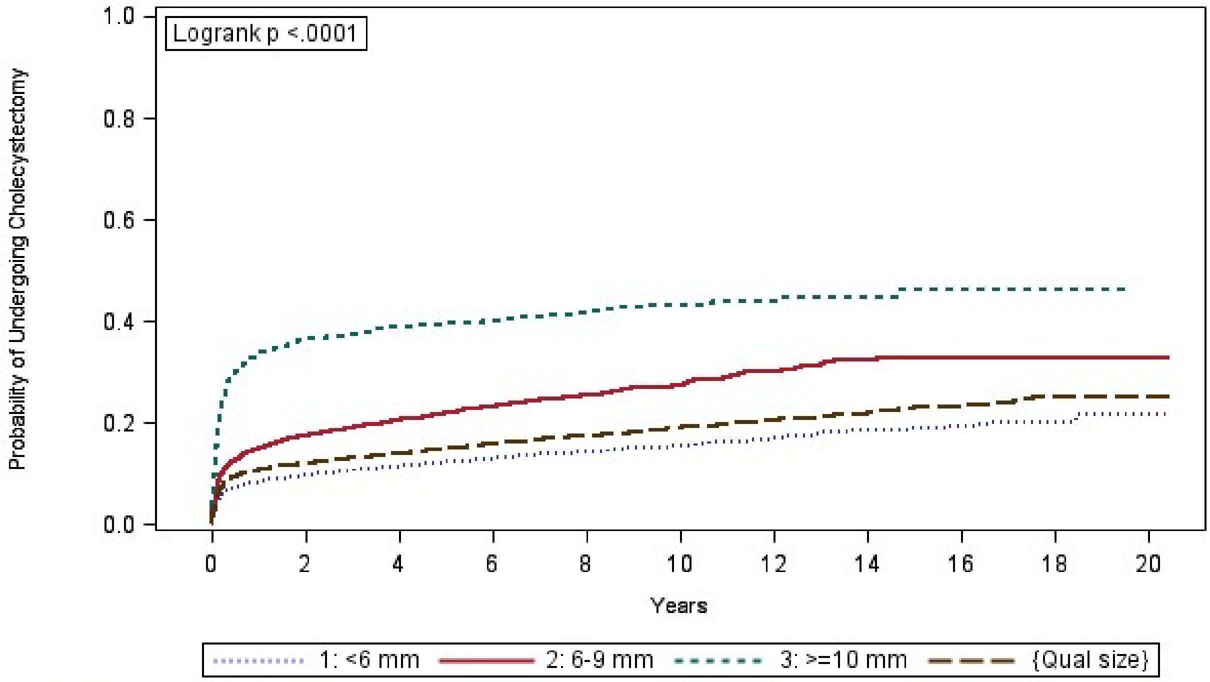

1: <6 mm	17531	11302	7212	4488	2823	1746	999	543	265	79	8
2: 6-9 mm	5912	3422	2202	1365	843	517	292	153	73	22	2
3: >=10 mm	2055	913	592	369	228	130	79	50	28	10	0
{Qual size}	10358	7519	6013	4759	3591	2541	1643	1029	551	219	38

© 2020 Szpakowski J-L et al. *JAMA Network Open.*
